# Case‐specific non‐linear finite element models to predict failure behavior in two functional spinal units

**DOI:** 10.1002/jor.24117

**Published:** 2018-08-21

**Authors:** Karlijn H. J. Groenen, Thom Bitter, Tristia C. G. van Veluwen, Yvette M. van der Linden, Nico Verdonschot, Esther Tanck, Dennis Janssen

**Affiliations:** ^1^ Orthopaedic Research Laboratory Radboud University Medical Center Radboud Institute for Health Sciences P.O. Box 9101 6500 HB Nijmegen The Netherlands; ^2^ Department of Radiotherapy Leiden University Medical Center P.O. Box 9600 2300 RC Leiden The Netherlands; ^3^ Laboratory for Biomechanical Engineering Department CTW University of Twente PO Box 217 7500 AE Enschede The Netherlands

**Keywords:** spine, bone metastases, finite element models, fracture prediction, failure behavior, quantitative computed tomography

## Abstract

Current finite element (FE) models predicting failure behavior comprise single vertebrae, thereby neglecting the role of the posterior elements and intervertebral discs. Therefore, this study aimed to develop a more clinically relevant, case‐specific non‐linear FE model of two functional spinal units able to predict failure behavior in terms of (i) the vertebra predicted to fail; (ii) deformation of the specimens; (iii) stiffness; and (iv) load to failure. For this purpose, we also studied the effect of different bone density–mechanical properties relationships (material models) on the prediction of failure behavior. Twelve two functional spinal units (T6‐T8, T9‐T11, T12‐L2, and L3‐L5) with and without artificial metastases were destructively tested in axial compression. These experiments were simulated using CT‐based case‐specific non‐linear FE models. Bone mechanical properties were assigned using four commonly used material models. In 10 of the 11 specimens our FE model was able to correctly indicate which vertebrae failed during the experiments. However, predictions of the three‐dimensional deformations of the specimens were less promising. Whereas stiffness of the whole construct could be strongly predicted (*R* ^2^ = 0.637–0.688, *p* < 0.01), we obtained weak correlations between FE predicted and experimentally determined load to failure, as defined by the total reaction force exhibiting a drop in force (*R* ^2^ = 0.219–0.247, *p* > 0.05). Additionally, we found that the correlation between predicted and experimental fracture loads did not strongly depend on the material model implemented, but the stiffness predictions did. In conclusion, this work showed that, in its current state, our FE models may be used to identify the weakest vertebra, but that substantial improvements are required in order to quantify in vivo failure loads. © 2018 The Authors. *Journal of Orthopaedic Research*® Published by Wiley Periodical, Inc. on behalf of Orthopaedic Research Society. J Orthop Res 36:3208–3218, 2018.

Patients with cancer and spinal bone metastases are at risk of developing pathological fractures and subsequent pain and neurological complications.^1^, ^2^ Hence, preventing pathological fractures is important to preserve quality of life and prevent debilitating complications.^3^, ^4^

Quantitative computed tomography (QCT)‐based finite element (FE) models are a promising tool for fracture risk assessment. These models can accommodate parameters related to the lesion, but can also cover additional aspects that play an important role in the assessment of the risk of fracture, such as the bone geometry, bone quality, and the daily loads applied to the bone.^5^ For example, the work by Whyne et al.^6^, ^7^, ^8^ and Tschirhart et al.^9^, ^10^, ^11^ studied the influence of tumor growth and loading scenario on precursors of burst fractures (e.g., load induced canal narrowing and vertebral bulge). These studies showed that, among others, tumor size, tumor location, pedicle involvement, bone density, loading rate, and spinal level affect the risk of initiating burst fractures. As such, the findings of these studies are important for improving the understanding of burst fracture mechanics in metastatically involved vertebrae and guiding future modeling efforts. These FE models, however, did not account for the effects of the heterogeneity in mineral density of the bone tissue, which is known to have a significant influence on fracture formation.^12^ Additional studies that did incorporate the patient‐specific geometry and material distribution have demonstrated that QCT‐based FE models can also simulate actual fractures, shown by accurate predictions of vertebral stiffness and strength, when compared to in vitro experiments.^13^, ^14^, ^15^, ^16^, ^17^, ^18^, ^19^

However, most FE models simulating actual fractures by implementing post‐yield behavior are confined to single vertebrae.^13^, ^14^, ^15^, ^16^, ^17^, ^18^, ^19^ Using single vertebrae induces simplified loading conditions. These simplified loading conditions are bound to introduce loading artifacts and may, therefore, be of less clinical relevance. A more physiological loading condition can be obtained by using two functional spinal units, consisting of three consecutive vertebrae and two intervertebral discs. This way, the middle vertebra is loaded via two intervertebral discs, the posterior elements, and the spinal ligaments; thereby transferring load as would happen under in vivo conditions. Using two functional spinal units, however, makes in vitro experiments and simulations more complex. This complexity is obviously one of the reasons why actual vertebral strength has not yet been predicted using FE models of two functional spinal units. Although some FE studies on metastatic spines did use two functional spinal units, they were parametric (and therefore did not account for bone density distribution) and focused on pre‐failure biomechanics rather than being patient‐specific and simulating actual fractures, and therefore are not able to predict actual strength.^9^, ^10^, ^20^

In addition, the mathematical relationships between bone density characteristics and material properties (hereinafter referred to as material model) describing bone behavior may have a significant effect on the FE predictions. We previously demonstrated that FE models of metastatic femurs can accurately predict both load to failure (*R* ^2^ = 0.93, *p* < 0.001) and fracture location, and can improve upon the strength prediction compared with experienced clinicians.^12^, ^21^ The material model of Keyak et al.^22^ was used in these simulations and includes post‐yield plastic behavior. Adopting material models based on vertebral bone might more accurately predict vertebral failure behavior. However, vertebral material models reported in the literature only partly describe mechanical behavior, for example, limiting to linking bone density to Young's modulus and/or yield stress, disregarding post‐yield behavior.^23^, ^24^, ^25^, ^26^, ^27^, ^28^, ^29^ In addition, these material models have been based on a broad range of bone densities. The resulting Young's moduli or yield stresses also vary considerably for a particular bone density.

The goal of this study was to develop a more clinically relevant, case‐specific non‐linear FE model of two functional spinal units able to predict failure behavior. When testing two functional spinal units it is not trivial that the vertebrae that failed during the in vitro experiments are correctly indicated by the FE models. Therefore, we firstly determined whether the FE models were able to correctly indicate which vertebrae failed during the experiments. Secondly, we investigated whether the deformation of the specimens was predicted correctly. Finally, we assessed whether the FE models were able to predict stiffness and load to failure. We, furthermore, studied the effects of different material models on the prediction of failure behavior by applying material models representing the broad spectrum reported in the literature.^22^, ^23^, ^24^, ^25^, ^26^ The overall aim of our project is to improve the clinical assessment of fracture risk in spinal metastatic bone disease.

## METHODS

The in vitro testing methodology, previously detailed,^30^ is hereby described in brief.

### Specimen Preparation

Three complete spines free from deformities and malignancies were obtained from fresh frozen cadavers (2 men, 1 woman; age 80–92 years). The specimens were obtained from the Department of Anatomy with ethical approval authorized by the Dean of the Radboud university medical center's Medical Faculty and in accordance with the human cadaveric protocol. Each spine was sectioned into four two functional spinal units (T6‐T8, T9‐T11, T12‐L2, and L3‐L5) while carefully preserving the facet capsules, intervertebral discs, and spinal ligaments. Since the ultimate goal of our project is to predict vertebral failure in spinal metastatic bone disease, we simulated metastatic lesions in the middle vertebral bodies in half of the specimens. The holes were created manually using hollow drills.^30^ The relative size of the lesion was kept constant between specimens: The depth of the lesion comprised 75% of the maximal vertebral body width and its diameter 55% of the maximal vertebral body height. The most posterior aspect of the lesion was aimed at 5 mm from the posterior vertebral body wall. The resulting defect was filled with 0.5% solution of agarose gel formulated to mimic the average material properties of lytic tumor specimens.^8^, ^31^ The removed core was dissected to isolate the cortical cap, which was reattached with polymethylmethacrylate (PMMA) to seal the lesion inside the vertebral body.^8^ The upper and lower vertebrae of each specimen were embedded in PMMA.

The specimens were subsequently immersed in water and scanned using QCT (Brilliance Big Bore, Philips Healthcare, Best, the Netherlands; 120 kVp, 300 mAs, slice thickness 1.0 mm, pitch 0.938, standard reconstruction, in plane resolution 0.977 mm). A solid calibration phantom (0, 50, 100, and 200 mg/ml calcium hydroxyapatite; Image Analysis, Columbia, KY) was scanned alongside. Bone density of the L1–L4 region of each specimen was measured in water using dual‐energy X‐ray absorptiometry (DEXA). After QCT and DEXA scanning, four and five tantalum pellets were inserted into the upper (spinous process and the left, right and anterior wall of the vertebral body) and lower vertebrae (left and right transverse processes and the left, right and anterior wall of the vertebral body), respectively, to allow motion detection using Rontgen Stereophotogrammetric Analysis (RSA).

### Mechanical Experiment

The specimens were fixated in a custom‐made testing jig inside an MTS machine (MTS DSTS 3301, 458.20 microconsole, MTS Systems Corporation, Eden Prairie, MN). Each specimen was mounted in the testing machine, such that the center of the middle vertebral body was aligned with the loading axis. The correct position of the specimen with respect to the loading pin was ensured using anteroposterior and mediolateral radiographs.^30^ The lower vertebra was constrained from translations and rotations in any direction. Free rotation in all directions was allowed for the upper vertebra. Following preconditioning, specimens were destructively tested in axial compression at 2 mm/min, while registering force and displacement (100 Hz). Specimens were loaded for at least 5 mm to ensure the occurrence of a fracture.^30^ RSA photographs were taken every 15 s to calculate the three‐dimensional (3D) position of the tantalum markers. To facilitate fracture detection and to capture the specimens’ post‐failure state, the specimens were fixated by embedding them in PMMA that was contained by a plastic bag, while the specimens were in the final loading position. Pre‐ and post‐experiment CT (Aquilion/CXL, Toshiba Medical, Otawara, Japan; resolution 0.6 × 0.6 × 0.6 mm) scans were evaluated by an experienced radiologist to determine the occurrence of a fracture and/or collapse in one of the vertebral bodies. Subsequently, we determined the load to failure from the force–displacement curves. The displacement in the loading direction of the upper vertebrae with respect to the lower vertebrae was calculated using the 3D positions of the tantalum markers, in order to account for play in the experimental set‐up. Force–RSA displacement curves were generated to determine the experimental stiffness.

### Finite Element Model

Based on QCT data we constructed FE models of all two functional spinal units. Geometric information for the vertebral bone and intervertebral discs (IVDs) was retrieved by segmenting the QCT images (Materialise Mimics 14.0, Materialise NV, Leuven, Belgium). All structures were meshed using four‐noded tetrahedral elements (mean edge length 2 mm) (HyperMesh 2017.1, HyperWorks, Altair Engineering, Inc. Troy, MI). The lesions were segmented separately from the specimens’ CT scans. The corresponding elements were subsequently deactivated during the simulations.

The IVD was discretized into a nucleus pulposus (NP) and annulus fibrosis (AF), with the NP area constituting approximately 40% of the total disc area.^32^, ^33^ Subsequently, the Herrmann formulation was applied to disc elements to account for the discs being nearly incompressible. Within the IVD, the NP and AF were modeled using hyperelastic Mooney‐Rivlin material models. The AF was furthermore modeled as a composite material with embedded rebar elements to represent the collagen fiber reinforcement. The fibers were placed circumferentially at ±20° with the transversal plane and only acted in tension. The mechanical properties of the NP and AF used in previous FE studies vary greatly.^32^, ^34^, ^35^, ^36^ The properties used in the current model are described in Table 1. Material properties for rather degenerated discs were chosen.^36^

**Table 1:** Material Properties Used for the Intervertebral Discs

	C10	C01	Bulk Modulus [MPa]	*E* [MPa]	*ν*
Nucleus	0.2	0.045	1.0e6		
Annulus fibrosis					
Ground substance	0.21	0.045	1.2e6		
Fibers				650	0.33

To allow for bone heterogeneity, ash density (*ρ*_ash_) of each element was determined from the QCT data using linear calibration.^22^ Subsequently, isotropic non‐linear material properties were assigned to each element based on four different Young's modulus–density and yield stress–density relationships previously empirically determined: (i) Keyak et al.^22^; (ii) Kopperdahl et al.^23^; (iii) Morgan et al.^24^, ^25^; and (iv) Ouyang et al.^26^ For Ouyang's material model, we modeled the Young's modulus both without (4a) and with (4b) the strain rate factor, which is often discarded in quasi‐static simulation studies.^37^ While the material model described by Keyak et al. is based on femoral bone, the others are based on vertebral bone. The vertebral material models were chosen as representatives for the high variations in both the bone densities used to define the material models and the resulting Young's modulus or yield stress.^38^ Most material models were based on apparent density (*ρ*_app_) or wet density (*ρ*_wet_) rather than on *ρ*_ash_. For these cases, *ρ*_app_ and *ρ*_wet_ were converted into *ρ*_ash_ using *ρ*_ash_ = 0.551 *ρ*_app_ and *ρ*_ash_ = 0.551 *ρ*_wet_, respectively.^38^, ^39^, ^40^ Moreover, only the material model reported by Keyak et al. included post‐yield plastic behavior. Hence, when studying the impact of the chosen material model, the Young's modulus and the yield stress were obtained from the varying material models, and post‐yield behavior as described by Keyak et al. was implemented in all models. In order to achieve continuous post‐failure behavior, the initial perfectly plastic phase, strain softening phase, and indefinite perfectly plastic phase were scaled according to the yield stress (Table 2).

**Table 2:** Bone Material Models Implemented in the Finite Element Models

		Young's Modulus (Original)	Yield Stress (Original)	Young's Modulus (*ρ*_ash_)	Yield Stress (*ρ*_ash_)	Scaling Factor for Post‐Failure Behavior
1	Keyak et al.^22^	*E* = 14,900 $\rho_{ash}^{1.86}$	*S* = 102 $\rho_{ash}^{1.80}$	*E* = 14,900 $\rho_{ash}^{1.86}$	*S* = 102 $\rho_{ash}^{1.80}$	–
2	Kopperdahl et al.^23^	*E* = 2,580 $\rho_{wet}^{1.34}$	*S* = 23.2 $\rho_{wet}^{1.60}$	*E* = 5,734 $\rho_{ash}^{1.34}$	*S* = 60.21 $\rho_{ash}^{1.60}$	*x* = 0.746 $\rho_{ash}^{-0.1112}$
3	Morgan et al.^24^, ^25^	*E* = 4,730 $\rho_{app}^{1.56}$	*S* = 37.1 $\rho_{app}^{1.74}$	*E* = 11,986 $\rho_{ash}^{1.56}$	*S* = 104.66 $\rho_{ash}^{1.74}$	*x* = 1.014 $\rho_{ash}^{-0.0334}$
4a	Ouyang et al.^26^ (excluding strain rate factor)	*E* = 2,383 $\rho_{app}^{1.88}$	*S* = 7.5 $\rho_{app}^{1.29}$	*E* = 7,307$\rho_{ash}^{1.88}$	*S* = 16.18 $\rho_{ash}^{1.29}$	*x* = 0.360 $\rho_{ash}^{-0.2834}$
4b	Ouyang et al.^26^ (including strain rate factor ϵ̇)	*E* = 2,383 $\rho_{app}^{1.88}$ϵ̇^0.07^	*S* = 7.5$\rho_{app}^{1.29}$	*E* = 7,307 $\rho_{ash}^{1.88}$ϵ̇^0.07^ ϵ̇ was assumed to be 1 · 10^−4^ s^−1^	*S* = 16.18 $\rho_{ash}^{1.29}$	*x* = 0.360 $\rho_{ash}^{-0.2834}$

**Figure 1:**
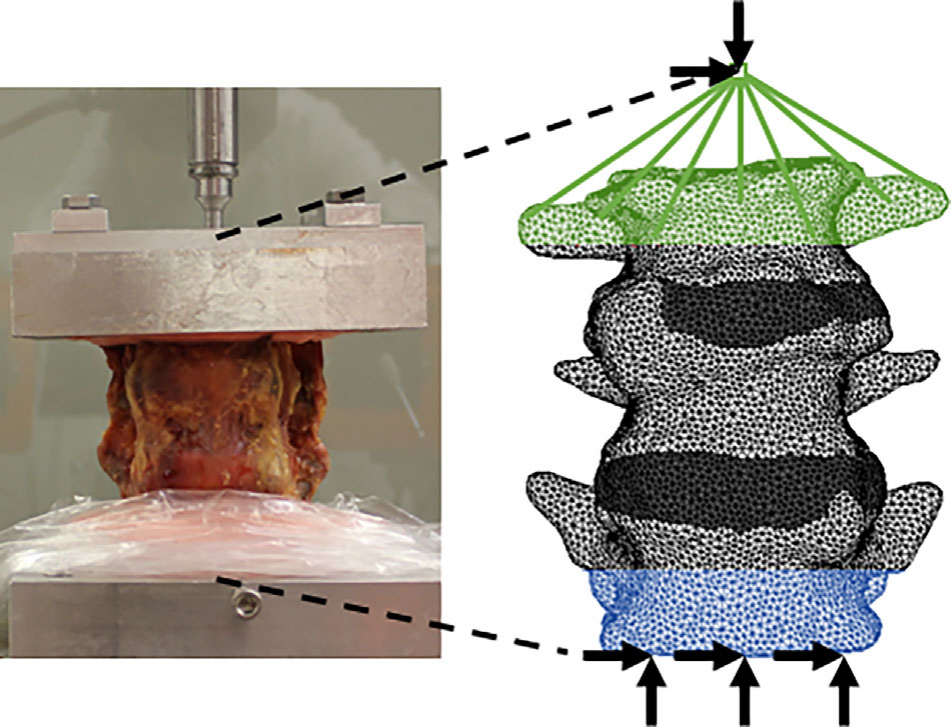
Diagrams showing the experimental setup (left) and the same conditions mimicked in the finite element models (right). The light shaded areas represent the vertebrae, the dark shaded areas represent the intervertebral discs. The displacement applied to the FE model was applied to the load node. The load node was constrained from translations in the horizontal plane. All surface nodes in the green colored area were connected to the load node using rigid links. The surface nodes in the blue colored area were constrained from translations and rotations in any direction.

The experimental boundary conditions were mimicked as realistically as possible in the FE simulations (Fig. 1). Touching contact without friction between facet joints was employed. The positions of the tantalum markers inserted into the vertebrae were copied to the FE model. In the FE simulations, we used a displacement‐controlled loading condition (0.1 mm/increment). The load was applied on a node that matched the point of load application during the experiments (load node). This node was connected to the upper vertebra via rigid links. The FE simulations were performed with MSC Marc (MSC.MARC2013r1; MSC Software Corporation, Santa Ana, CA). The incremental displacement in the loading direction of the upper vertebra with respect to the lower vertebra was registered via the virtual RSA markers in the upper and lower vertebrae. The total reaction force in the loading direction was defined as reaction force in the load node due to the prescribed displacement. Structural fracture was assumed to occur when the total reaction force exhibited a drop in force. Stiffness was determined from the reaction force–RSA based displacement curve. The location of failure was defined by elements that plastically deformed^22^ when the load to failure was reached. For a more detailed analysis of fracture location, we assessed the experimentally determined and FE predicted 3D deformation of the specimen. To this end, contours of the vertebrae post‐experiment and post‐FE were qualitatively examined with respect to pre‐experimental vertebral contours. Contours were obtained from stereolythography (stl) files obtained from segmentations of the pre‐ and post‐experimental CT scans, and FE model in the post‐failure state. The iterative closest point (ICP) method was used to register stl files based on the lower vertebrae.

### Data Analysis

Specimens in which multiple vertebrae failed during the experiments were excluded from further analyses, since our experimental test setup did not allow for assessing the order of vertebral failure. The vertebrae within the two functional spinal units that failed in the FE simulations and corresponding experiments were qualitatively compared. In addition, the deformation of the experimental specimens was qualitatively compared to the deformation predicted by the FE models. In case fracture was predicted to occur in the incorrect vertebra, the corresponding simulation was excluded from stiffness and load to failure analyses. We used linear regression analysis to evaluate relations between the predicted and measured stiffness and load to failure. Statistical analyses were performed in MATLAB (Release 2016b, The Mathworks, Inc., Natick, MA). Results were considered significant if *p* < 0.05.

## RESULTS

### In Vitro Experiments

Data from all specimens and experimental results are summarized in Table 3. Radiological examination of the pre‐ and post‐experimental CT scans showed that 11 specimens failed in only one vertebra and were included for further analyses. Of these, 10 specimens fractured in the middle vertebrae, whereas one fractured in the lower vertebra (B2).

**Table 3:** Overview of the Tested Specimens and Experimental Results

Sex (M/F)	Age at Death [y]	Weight [kg]	Length [cm]	BMI [kg/m^2^]	*T*‐Score	Bone Quality	Specimen		Lesion (L)/Intact (I)	Failed Vertebra(e) Upper (U); Middle (M); Lower (L)	Stiffness [N/mm]	Load to Failure [N]
F	80	44	157	17.9	−3.3	Osteoporotic	A1	T6‐T8	I	M	7,072	3,232
							A2	T9‐T11	L	M	7,535	2,573
							A3	T12‐L2	L	M	2,206	2,339
							A4	L3‐L5	I	M	4,078^b^	3,929^b^
M	92	87	187	24.9	−0.7	Normal	B1	T6‐T8	L	M	4,799	3,307
							B2	T9‐T11	I	L	4,552	2,702
							B3	T12‐L2	I	M	4,176	3,294
							B4	L3‐L5	L	M	2,359	2,474
M	80	55	163	20.7	−3.1	Osteoporotic	C1	T6‐T8	I	M^a^	3,251	1,780
							C2	T9‐T11	L	M	1,462	1,386
							C3	T12‐L2	L	M	1,017	1,275
							C4	L3‐L5	I	M + L	–	–

^a^ The lower vertebra had only slightly collapsed, whereas the middle one showed considerably more damage. Therefore, this specimen was analyzed as if failure occurred in the middle vertebra.

^b^ Whereas the experiments showed failure in the middle vertebra, the FE models predicted the lower vertebra to fail. Therefore, specimen A4 was excluded from stiffness and load to failure analyses.

### FE Simulations

The 11 experiments in which one vertebra failed were all simulated by the FE models. For each specimen, we ran five simulations comprising the different material models, resulting in 55 simulations. One simulation had convergence problems before reaching failure (Kopperdahl et al.: specimen B3). However, the force–displacement curve already started to deflect. Therefore, this simulation was included in the load to failure analysis.

### Identification of the Vertebra to Fail

**Figure 2:**
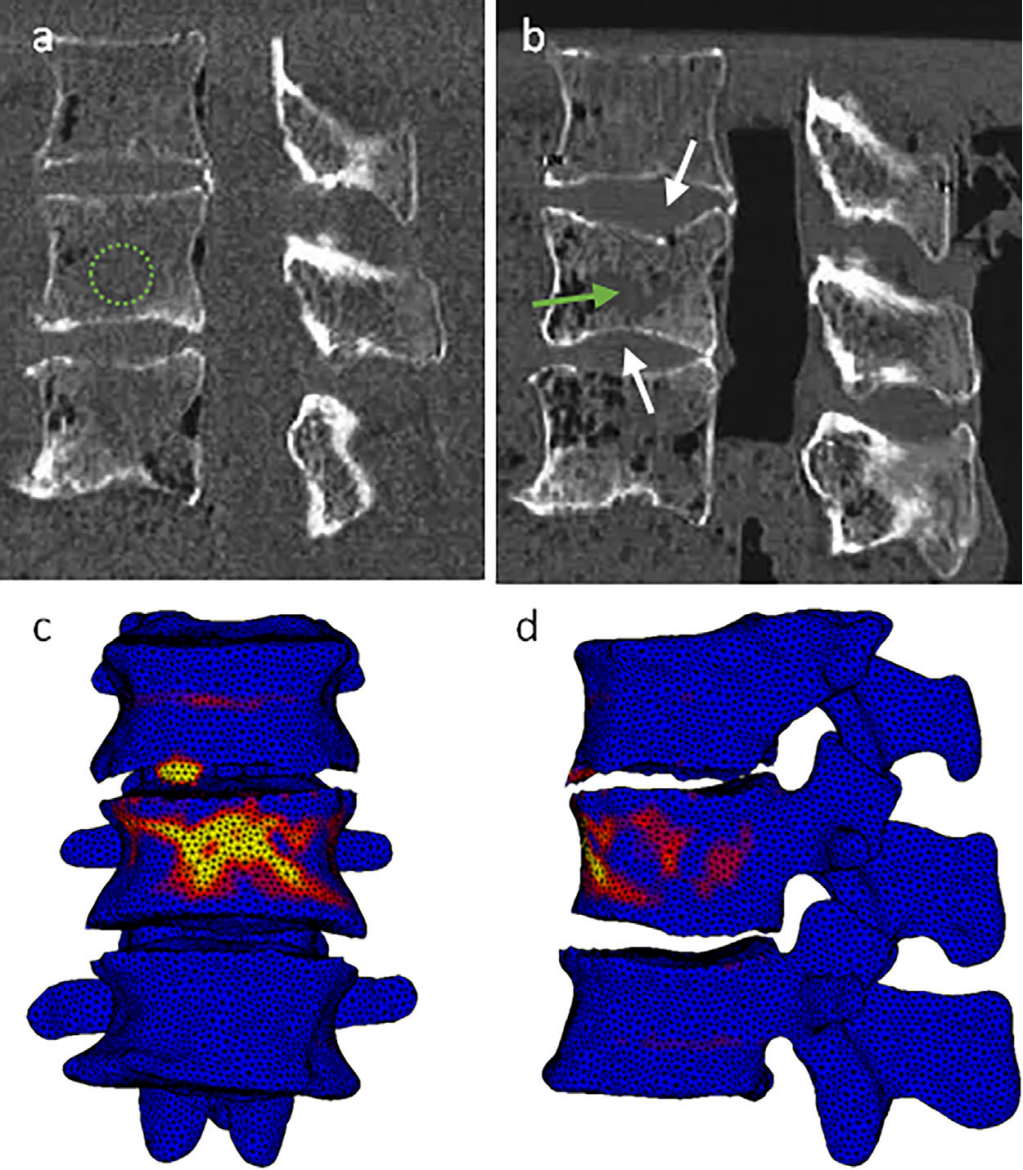
Finite element model predictions of a fracture location, showing areas of plastic deformity (indicated in red/orange/yellow), with experimental CT scans showing failure in the corresponding vertebra. (a) Pre‐experimental CT scan (simulated lesion indicated by the green circle); (b) post‐experimental CT scan with the arrows indicating endplate failure (simulated lesion indicated by the green arrow); (c) plastic deformation located mainly in the middle vertebral body shown in anterior view; (d) plastic deformation located mainly in the middle vertebral body shown in lateral view. Specimen shown: A3.

The material model implemented did not affect which vertebra was predicted to fail (i.e., which vertebra was identified as weakest). Furthermore, in 10 out of 11 specimens the vertebra to fail was correctly indicated by the FE model (Fig. 2). This was not necessarily the middle vertebra, since in specimen B2 both the experiment and FE simulations indicated the lower vertebra as the weakest one. Whereas in specimen A4 the middle vertebra failed during the experiment, the lower one was predicted to fail by the FE simulations. Consequently, specimen A4 was dismissed from further analyses.

### Specimen Deformation

**Figure 3:**
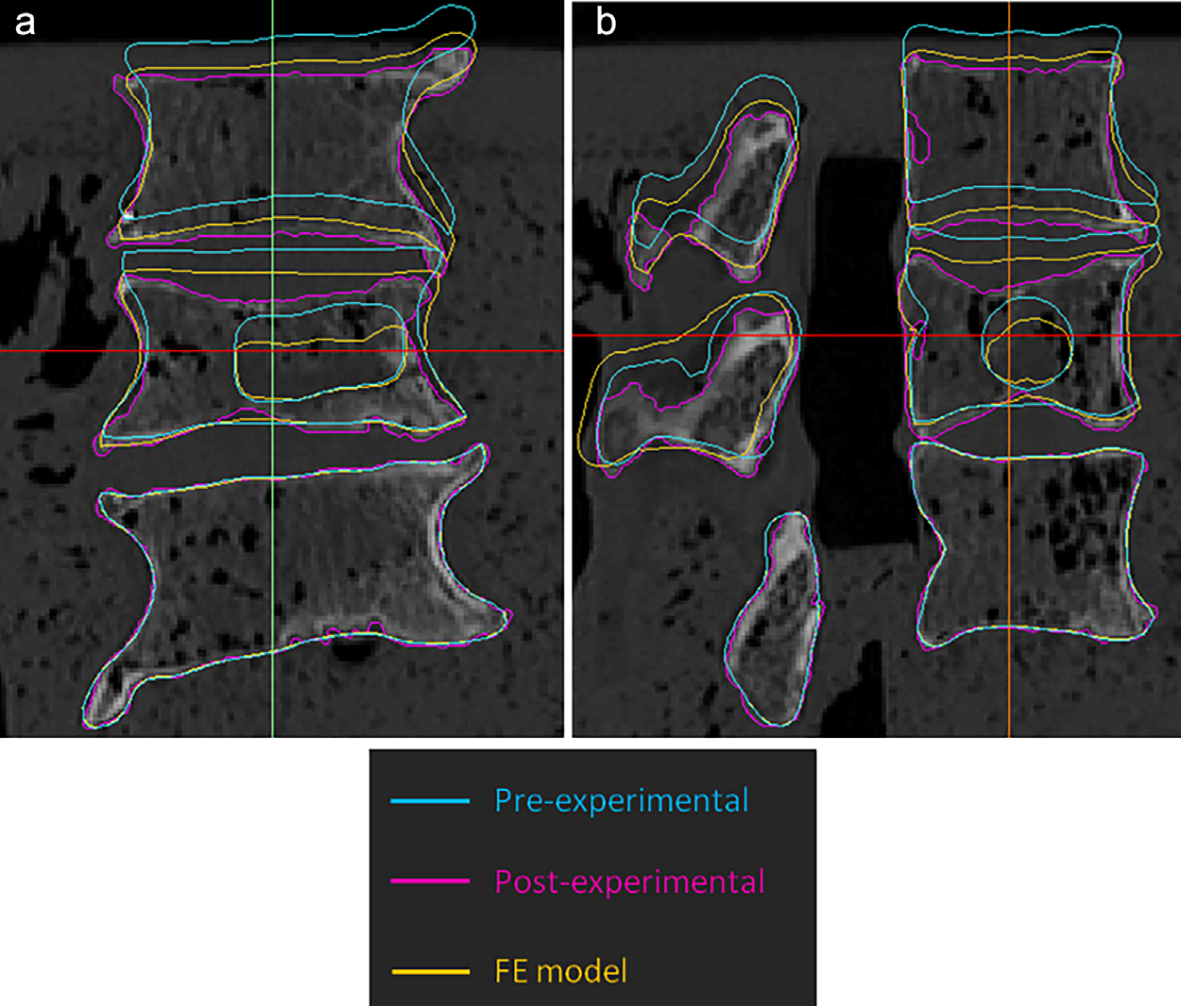
Vertebral contours comparing experimental and FE predicted 3D deformation of the entire specimen in the (a) mid‐frontal view and (b) mid‐sagittal view. This representative specimen shows that in most cases deformation of the lower vertebrae was correctly predicted by the FE models, illustrated by overlapping contours of the pre‐ and post‐experimental contours. In addition, in the experiments often substantial fractures were seen in the endplates, whereas this was not observed in the simulations. Specimen shown: A3.

When comparing the measured experimental deformation to the deformation predicted by the FE models, it appeared that most models did not converge to a point where the full experimental displacement was reached, as shown by the difference between the post‐experimental and predicted position of the upper vertebra (Fig. 3). Furthermore, in case deformation barely occurred in the lower vertebra, this was predicted correctly by the FE models: The contours of the pre‐ and post‐experimental segmentations as well as of the FE model were overlapping. While in the experiments often substantial fractures were seen in the endplates, this was not observed to such a large extent in the simulations, where plasticity mainly occurred transversally in the vertebral bodies (Fig. 2). In addition, the material model did not substantially affect the predicted plasticity pattern.

### Stiffness

**Figure 4:**
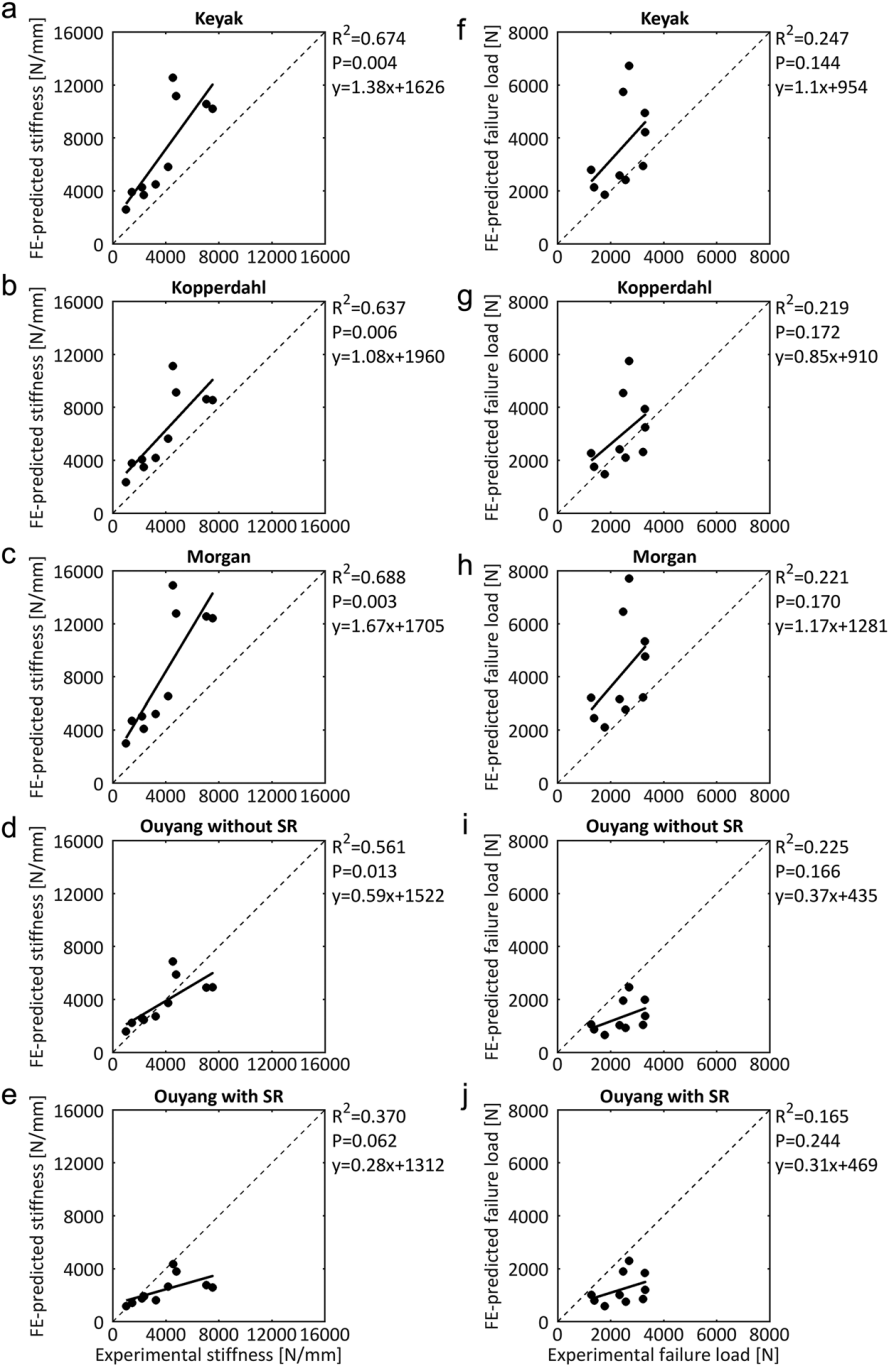
Linear regression of the experimentally measured stiffness versus the stiffness predicted by the finite element (FE) model (a–e) and experimentally measured load to failure versus the load to failure predicted by the FE model (f–j) for the different implemented material models. SR, strain rate factor.

There was a significant, strong correlation between the experimental and FE predicted stiffness when the material models by Keyak et al. (*R* ^2^ = 0.674, *p* < 0.01), Kopperdahl et al. (*R* ^2^ = 0.637, *p* < 0.01), and Morgan et al. (*R* ^2^ = 0.688, *p* < 0.01) were implemented (Fig. 4). Of these, no one was clearly superior. For Ouyang et al.'s material model stiffness could weakly or moderately be predicted, depending on whether or not the strain rate factor was included (without strain rate factor: *R* ^2^ = 0.561, *p* < 0.05; with strain rate factor: *R* ^2^ = 0.370, *p* > 0.05) (Fig. 4). While the material models by Keyak et al., Kopperdahl et al., and Morgan et al. overpredicted the stiffness in all specimens, it was underestimated in most specimens when Ouyang et al. with strain rate factor was applied (Fig. 4).

### Load to Failure

The load to failure could be weakly predicted: *R* ^2^ ranged from 0.219 to 0.247 (*p* > 0.05) (Fig. 4). The slopes of the regressions by Keyak et al. (slope = 1.1), Kopperdahl et al. (slope = 0.85), and Morgan et al. (slope = 1.17) were closer to one than the slopes of simulations by Ouyang et al. (without strain rate factor: Slope = 0.37; with strain rate factor: Slope = 0.31) (Fig. 4). None of the models of Keyak et al., Kopperdahl et al., or Morgan et al. could be appointed as being superior. When implementing the material model by Ouyang et al. with strain rate factor, the prediction of load to failure was very weak (*R* ^2^ = 0.165, *p* > 0.05). Furthermore, the FE models based on material models by Keyak et al. and Morgan et al. overestimated the load to failure, while those based on Ouyang et al., irrespective of the strain rate factor, underestimated the load to failure (Fig. 4). Implementing Kopperdahl et al.'s material model resulted in some specimens to be overestimated and some to be underestimated.

## DISCUSSION

This study aimed to develop a case‐specific non‐linear FE model of two functional spinal units able to predict the failure behavior in terms of (i) the vertebra predicted to fail; (ii) the deformation of the vertebrae; (iii) stiffness; and (iv) load to failure. For this purpose, we also studied the effect of different material models.

To obtain a more realistic biomechanical environment, we performed experimental tests and created FE models of two functional spinal units instead of isolated vertebrae. Although we are convinced that this approach is more clinically relevant than considering single vertebrae, it also generated additional challenges. When testing three consecutive vertebrae, it is not obvious that the vertebra to fail is correctly predicted by the FE models. Nevertheless, in our study all but one predictions were accurate. When examining the predicted fracture location in more detail, however, it appeared that the predicted specimens’ 3D deformation did not completely match with the measured deformation, particularly in cases of endplate failure. We also showed that most FE models did often not converge to a point where the full experimental displacement was reached. Possibly, the predicted and experimentally measured 3D deformation would have corresponded better when the convergence problems are solved. Further improvements of the model in this respect are required.

There have been several attempts to predict compressive stiffness of single vertebrae, with a wide range of predictive capability: *R* ^2^ = 0.50–0.81.^15^, ^16^, ^17^, ^41^, ^42^, ^43^, ^44^ We reported on structural stiffness of models comprising three vertebrae and two IVDs. Despite the increased complexity of our model, we obtained similar results when the bone material models by Keyak et al., Kopperdahl et al., or Morgan et al. were implemented.

In contrast, the current study could not predict vertebral load to failure as accurately as previous studies. While previous QCT based FE studies on single vertebra reported strong to very strong correlations (*R* ^2^ = 0.76–0.96^13^, ^14^, ^15^, ^16^, ^17^, ^19^, ^41^, ^44^, ^45^), the correlations we obtained were weak, irrespective of the material model obtained. However, comparisons are complicated due to the wide spread in the criteria used to indicate (or quantify) failure, and in some cases these criteria were fitted to match the experimental data. In the current study, we simulated post‐yield plasticity, thereby modeling the full failure process, rather than fitting an “arbitrary” FE strength criterion to the experimental strength values. Our attempt to more realistically simulate vertebral failure seems, for now, not yet sufficient for obtaining an accurate prediction. However, we do believe that by simulating the actual failure process we will eventually obtain a more robust and true prediction of vertebral failure than in cases where failure criteria are used which may not be applicable under varying clinical circumstances.

The poorer strength prediction may be due to several differences between previous studies and the current models, in terms of modeling approach, medical imaging data that were used for the models, and the loading configuration. Firstly, previous models have been based on single vertebrae, thereby neglecting the posterior elements including the facet joints, while these are known to carry up to 33% of the axial load.^46^ Conversely, the current study included three consecutive vertebrae with the IVDs. Loading via IVDs has shown to increase the strain levels in vertebral body, especially in the endplate.^47^ Lu et al.^36^ and Maquer et al.^48^ investigated whether strength predictions computed from HR‐pQCT based FE models of human vertebral bodies were influenced by the choice of boundary condition (PMMA vs. IVD). Both studies suggested that adding the IVDs to vertebral body models is not very contributive until fully validated patient‐specific IVD models become available. In the current study a simplified disc was implemented, with similar properties as the IVDs modeled by Maquer et al. and Lu et al. Possibly, the mechanical representation of the IVD in our model is too simplistic, resulting in nonrealistic deformation, particularly in the endplate. This might also explain why the FE models did not show endplate collapse, whereas we did find that in the experiments.

The lack of success in predicting endplate failure might indicate that we incorrectly modeled the intervertebral disc. The absence of a thorough validation of the discs’ behavior is a flaw in the present study. However, we have performed several sensitivity analyses on the disc material parameters. The effect of varying these disc material parameters on the resulting force–displacement curves appeared to be minimal. For example, changing the stiffness of the fibers by 30% resulted in a 2% change in overall stiffness of the full model, and a 0.5% change in strength. Moreover, increasing the C01 coefficient of both the annulus and the nucleus did not significantly affect the force response. We also varied the fiber angle (between 0° and 90° with the transversal plane). The difference in failure force when 10° and 90° angels were used was about 7%. Changing the fiber angle from 20° to 30° resulted in a 2% change in failure load and 4% change in stiffness. Nevertheless, future work should also be directed to a more thorough validation of the disc properties. This also implicates that appropriate measurements on disc deformation should be included when performing the in vitro experiments. In the present study, we considered using the RSA markers for validation purposes. However, given the small displacements measured, even within the whole construct, together with the relatively course CT scan would result in inaccurate measurements.

Furthermore, in proceeding from the failure behavior of a single vertebra to subject‐specific two functional spinal units, a major difference is the effect of the intervertebral discs on the loading distributions between vertebrae. Incorporating case‐specific disc models as well as disc degeneration (given the fact that the donors were +80 years old) might therefore be important issues. The geometry of the discs was obtained from CT scans, thereby taking the case‐specific and degenerated geometry into account, as demonstrated by the small thickness of the discs and the locations of bone‐on‐bone contact in our models. In contrast, the discs’ distinction between annulus and nucleus, and the discs’ material properties were not case‐specific. Determining the exact quality of the disc material and consequently its mechanical properties based on CT or MRI imaging currently is, however, challenging. Moreover, we propose that the limited thickness and the extent of bone‐on‐bone contact is most likely the reason why the changes in material properties in our sensitivity analyses did not have a substantial effect on the fracture force or fracture location in our models. Nevertheless, future research should be aimed at improving the disc's material model and interactions between discus and vertebrae, preferably in specimens of a better quality, where the discs will have a larger effect.

Previous research has demonstrated the importance of modeling the spine using poro‐elasticity in order to represent the mechanics of a burst fracture in the metastatic spine.^7^ Poro‐elasticity was, however, not implemented in our model. In both the experimental and FE model developed in the present study, the loading rate was rather low and considered to be quasi‐static. Therefore, pressurization of the lesion creating high stresses in the vertebra, leading to failure, most likely did not play a big role. The quasi‐static loading, however, might not be clinically relevant, as in a clinical setting vertebral fractures might occur under the application of a fast load. In these cases, the use of multiphasic models might be more appropriate.

The inaccurate prediction of endplate failure may also be due to the fact that CT scans were used with clinical settings instead of high‐resolution CT scans. Similar resolution scans^13^, ^16^ have previously been used to successfully predict vertebral strength in single vertebra models (*R* ^2^ = 0.8–0.86). However, the endplate and cortex of the vertebra are quite thin, with a thickness of approximately 0.1–1.25 mm.^49^, ^50^, ^51^ Due to partial volume effects, the endplates and cortical shell may not have been captured properly in the FE model. An alternative approach, as is adopted by others,^14^, ^15^, ^45^, ^52^ could be to add an extra shell to the vertebral body, representing the cortex and/or endplates. Chevalier et al.^42^ demonstrated that adding an explicit cortex stiffened and strengthened their high resolution QCT models. Since most of our models already overestimated stiffness and strength, adding a shell would most probably not improve our results, particularly since our models already had difficulties capturing endplate fractures.

Furthermore, we performed only compression testing, while the loading conditions of daily living also include flexion and other loading modes. From a practical point of view, a compression test is easier to execute than tests that include bending, especially when using two functional spinal units. In our experimental set‐up (and in our models), however, the specimens were allowed to “pivot” around the load application point, thereby inducing forward or lateral bending motions. Although the performance of FE models comprising single vertebrae is well known for uniform compression, the ability to predict vertebral strength under non‐uniform loading conditions is less clear. The predictive capacity in axial compression might not be indicative of its capacity in bending, as it was previously shown that it is difficult to predict the strength of isolated vertebral bodies in anterior bending (*R* ^2^ = 0.34–0.40).^53^ However, other FE models were able to accurately predict vertebral strength when anterior wedge shape fractures were experimentally induced in single vertebral bodies (*R* ^2^ = 0.77–0.79).^18^, ^44^ Given these inconsistencies in the literature, further research into the effect of loading configuration is required.

Moreover, we showed that the correlation between predicted and experimental fracture loads did not strongly depend on the material model, but the stiffness predictions did. For both stiffness and load to failure, FE models based on the material models by Keyak et al.^22^ and Morgan et al.^24^, ^25^ resulted in overpredictions of the experimental measurements, whereas models based on Ouyang et al.'s equations^26^ led to underpredictions. Similarly, Silva et al.^54^ predicted fracture load and stiffness in midsagittal sections of human vertebral bodies by implementing modulus and strength from density, based on Keller^27^ and Kopperdahl and Keaveny (in the paper referred to as “personal communication, 1995”). They also found that the predicted fracture loads did not strongly depend on the material density‐elastic property relations, whereas the stiffness values were highly affected by the material of choice. Stiffness was typically underestimated by a factor of 2–3 on the basis of the Keller relations and was over estimated by a factor of 2–3 on the basis of the Kopperdahl and Keaveny relations.

This work shows that, in its current state, our FE models may be used to identify the weakest vertebra, but that substantial improvements are required in order to quantify in vivo failure loads.

First, the FE model might profit from more realistic IVD models.^36^, ^48^ In contrast to the bone material behavior, the IVD properties used in this study were not case‐specific but obtained from the literature. However, both the type of material model and values for coefficients used in previous FE studies highly varied.^32^, ^34^, ^35^ The effect of these varying parameters on predictions of vertebral stiffness and/or bone strength is not well investigated. Therefore, effort could be put in determining (case‐specific) mechanical properties of IVD tissue, and, subsequently, in investigating how implementing these properties in FE models affects the failure behavior of both single vertebra and two functional spinal units. In addition, gaining more insight into the effect of IVD properties on endplate failure would be valuable, as endplate failure could not be captured correctly by the current FE model. For this reason, emphasis should also be put on further characterizing and adequately simulating the endplates’ mechanical properties.

In addition, in case of sufficient resources and anatomical specimens, it would be interesting to combine testing of two functional spinal units with single vertebra tests. In this way, the validity of the material models can be better tested.

Lastly, whereas in the experiments soft tissues, including the spinal ligaments and facet capsules, were left intact, these structures were not accounted for in the FE simulations. Spinal ligaments may contribute to the specimens’ stiffness and strength, especially when moving in flexion, extension, or lateral bending. As we allowed the specimens to pivot around the load application point, such movements could occur. Adding ligaments and facet capsules to the FE model provides loading conditions being more realistic and better mimicking the experimental conditions, which potentially results in a better predictive capacity of the FE model.

## CONCLUSION

In conclusion, whereas the FE model was able to correctly indicate which vertebrae failed during the experiments, it had difficulties predicting the 3D deformation of the specimens. In addition, stiffness could be strongly predicted by our model, but we obtained weak correlations between FE predicted and experimentally determined vertebral strength. Therefore, this work showed that, in its current state, our FE models may be used to identify the weakest vertebra, but that substantial improvements are required in order to quantify in vivo failure loads.

## AUTHORS' CONTRIBUTIONS

All authors substantially contributed to research design, data acquisition, analysis, and interpretation of data; substantially contributed to drafting the paper or revising it critically; and approved the submitted and final version of the manuscript.
